# Gout in immigrant groups: a cohort study in Sweden

**DOI:** 10.1007/s10067-016-3525-1

**Published:** 2017-01-13

**Authors:** Per Wändell, Axel C Carlsson, Xinjun Li, Danijela Gasevic, Johan Ärnlöv, Martin J Holzmann, Jan Sundquist, Kristina Sundquist

**Affiliations:** 1 Department of Neurobiology, Care Sciences and Society, Division of Family Medicine, Karolinska Institutet, Alfred Nobels Allé 23, SE-141 83 Huddinge, Sweden; 2 Department of Medical Sciences, Cardiovascular Epidemiology, Uppsala University, Uppsala, Sweden; 3 Center for Primary Health Care Research, Lund University, Malmö, Sweden; 4 Usher Institute of Population Health Sciences and Informatics, College of Medicine and Veterinary Medicine, University of Edinburgh, Edinburgh, UK; 5 Department of Emergency Medicine, Karolinska University Hospital, Huddinge, Sweden; 6 Department of Internal Medicine Solna, Karolinska Institutet, Stockholm, Sweden

**Keywords:** First-generation immigrants, Gender, Gout, Neighbourhood, Second-generation immigrants, Socio-economic status

## Abstract

Our aim was to study the association between country of birth and incidence of gout in different immigrant groups in Sweden. The study population included the whole population of Sweden. Gout was defined as having at least one registered diagnosis in the National Patient Register. The association between incidence of gout and country of birth was assessed by Cox regression, with hazard ratios (HRs) and 95% confidence intervals (95% CI), using Swedish-born individuals as referents. All models were conducted in both men and women, and the full model was adjusted for age, place of residence in Sweden, educational level, marital status, neighbourhood socio-economic status and co-morbidities. The risk of gout varied by country of origin, with highest estimates, compared to Swedish born, in fully adjusted models among men from Iraq (HR 1.82, 95% CI 1.54–2.16), and Russia (HR 1.69, 95% CI 1.26–2.27), and also high among men from Austria, Poland, Africa and Asian countries outside the Middle East; and among women from Africa (HR 2.23, 95% CI 1.50–3.31), Hungary (HR 1.98, 95% CI 1.45–2.71), Iraq (HR 1.76, 95% CI 1.13–2.74) and Austria (HR 1.70, 95% CI 1.07–2.70), and also high among women from Poland. The risk of gout was lower among men from Greece, Spain, Nordic countries (except Finland) and Latin America and among women from Southern Europe, compared to their Swedish counterparts. The increased risk of gout among several immigrant groups is likely explained by a high cardio-metabolic risk factor pattern needing attention.

**Electronic supplementary material:**

The online version of this article (doi:10.1007/s10067-016-3525-1) contains supplementary material, which is available to authorized users.

## Introduction

Gout is the most common inflammatory arthritis where monosodium urate crystals are deposited in joints and soft tissues. Individuals with gout experience acute attacks of excruciating pain; and, if left untreated, gout may lead to debilitating complications such as chronic joint damage and renal insufficiency [1]. All these contribute to the poor patient’s health-related quality of life [2].

In addition, gout is also associated with different metabolic conditions, such as insulin resistance [3], the metabolic syndrome and diabetes mellitus [4]. Besides, there is also a strong relationship between gout and hypertension and with antihypertensive diuretic treatment [5, 6], especially with thiazide diuretics [7, 8], and with other cardio-vascular diseases such as chronic heart failure [5] and chronic kidney disease [9]. Gout is also associated with an increased mortality risk, mainly through the increased risk of cardio-vascular diseases, including coronary heart disease [10].

As a metabolic disorder, gout is associated with several established risk factors according to epidemiological studies [11]: genetic factors, excess alcohol consumption [12] and with a purine-rich diet, especially with high rate of hypoxanthine, i.e. diets with animal meats, fish meats, organs such as liver and fish milt and yeast [13].

Recent review of epidemiological evidence has indicated that gout has risen worldwide over the last few decades [1], tailing the obesity epidemic [11]. Besides, the clinical picture of gout seems to have become more complex [11]. However, there is a large variation in the prevalence data of gout. Evidence from a recent review and meta-regression indicates that age, sex, continent on which study was performed and the case definition of gout accounted for the large variation in gout prevalence across studies [9]. Indeed, ageing is a risk factor for gout in both sexes; however, gout is more prevalent in men than in women [14, 15]. Furthermore, there are significant differences in prevalence of gout across the continents where the highest prevalence with estimates of >10% has been observed in Oceanian countries and a high prevalence of 1–4% in most countries in North America and Western Europe [16]. Lower prevalence has been observed in former Soviet Union regions, Guatemala, Philippines, Malaysia, Iran, rural Turkey, Saud Arabia and African countries.

In Sweden, almost one fifth of the population is foreign born, and immigration to Sweden increases with each year [17]. Describing and better understanding of disparities in gout among immigrants are of great interest both for the health care and the society in general for possible preventive actions. The aim of this study was to explore the risk of being diagnosed with gout among first- and second-generation immigrants in Sweden.

## Methods

### Design

The dataset used in this study was retrieved from governmental national registers such as the Total Population Register (TPR) and the National Patient Register (NPR) that contain longitudinal information on the entire population of Sweden for a period of 40 years. The TPR contains comprehensive nationwide individual-level data as well as data on neighbourhood socio-economic status (SES). The Swedish nationwide population and health care registers have exceptionally high completeness and validity [18]. Individuals were tracked using their personal identification numbers, which are assigned to each resident of Sweden. These identification numbers were replaced with serial numbers to provide anonymity. Subjects of 45 years of age and older were included in the study. The follow-up period ran from January 1, 1998 until hospitalization/outpatient treatment of gout at death, emigration or December 31, 2012, whichever came first.

### Study population and co-morbidities

This study included the whole Swedish population. Country of birth was registered, and we included 10 regions (Nordic countries, Southern Europe, Western Europe, Eastern Europe, Baltic countries, Central Europe, Africa, North America, Latin America and Asia) and 27 countries (Supplementary Table 1). Countries with less than 10 observed cases of gout were not analysed separately. The second-generation immigrants were defined according to the Swedish Multi-generation Register, based on their parental immigrant’s information.

The reference population in the analysis for the second-generation immigrants was Swedes in at least two generations that are adults 45 years of age and older born in Sweden and with both father and mother born in Sweden.

Patients with diagnosed gout were identified by the presence of the ICD-10 code (tenth version of the WHO’s International Classification of Diseases) for gout (M10) in the National Patient Register. Patients with gout diagnosed before 1998, i.e. during the years 1987–1997 (according to ICD-9 1987–1996 and ICD-10 1997) were excluded.

We also identified co-morbidities according to ICD-10 for the following diagnoses: hypertension I10–I19, coronary heart disease (CHD) I20–I25, heart failure I50, stroke I60–I69, diabetes E10–E14, obesity E65–E68, alcoholism and related disorders F10 and K70 and chronic obstructive pulmonary disease (COPD) J40–J47.

### Outcome variable

Gout ICD-10 code M10.

### Demographic and socio-economic variables

*Sex*: men and women.

*Age* was used as a continuous variable in the analysis.

*Educational attainment* was categorized as ≤9 years (partial or complete compulsory schooling), 10–12 years (partial or complete secondary schooling) and >12 years (attendance at college and/or university).

*Geographic region of residence* was included in order to adjust for possible regional differences in hospital admissions and was categorized as (1) large cities, (2) southern Sweden and (3) northern Sweden. Large cities were defined as municipalities with a population of >200,000 and comprised the three largest cities in Sweden: Stockholm, Gothenburg and Malmö.

### Neighbourhood socio-economic status

The neighbourhoods were derived from small-area market statistics (SAMS), which were originally created for commercial purposes and pertain to small geographic areas with boundaries defined by homogenous types of buildings. The average population in each SAMS neighbourhood is approximately 2000 people for Stockholm and 1000 people for the rest of Sweden. A summary index was calculated to characterize neighbourhood-level deprivation. The neighbourhood index was based on information about female and male residents aged 20 to 64 years, because this age group represents those who are among the most socio-economically active in the population (i.e. a group that has a stronger impact on the socio-economic structure in the neighbourhood compared to children, younger women and men and retirees). The index was based on the following four variables: low educational status (<10 years of formal education); income from all sources, including interest and dividends, that is <50% of the median individual income; unemployment (excluding full-time students, those completing military service and early retirees); and receipt of social welfare. The index was categorized into three groups: more than one standard deviation (SD) below the mean (high SES or low deprivation level), more than one SD above the mean (low SES or high deprivation level) and within one SD of the mean (middle SES or deprivation level) [19], with neighbourhood status classified as high, middle or low SES or on low, middle and high deprivation index [20].

### Statistical analysis

Baseline subject characteristics were presented for population samples when estimating incidence rates of gout among first-generation immigrants and among second-generation immigrants.

Cox regression was used for estimating the risk for gout in different immigrant groups compared to Swedish born as referents. Time was from January 1, 1998, or immigration date until hospitalization/outpatient treatment of gout at diagnosis, death, emigration or the end of the study period on December 31, 2012.

All analyses were stratified by sex. Four models were used: model 1 was adjusted for age and region of residence in Sweden; model 2 was adjusted for age, region of residence in Sweden, educational level and marital status; model 3 was as model 2 with the addition of neighbourhood SES; and model 4 was as model 3 with the addition of co-morbidities. As a sensitive analysis, we also analysed hazard ratios (HRs) for first-generation immigrants with exclusion of immigrants who moved to Sweden within 5 years of follow-up with full adjustment according to model 4.

The study was approved by the local ethical vetting board at the Karolinska Institutet (reference number 12/00 EPN Huddinge at 6 March 2000, approval of addition at 18 November 2002).

## Results

Table 1 presents the characteristics of the included samples for first- and second-generation immigrants. Among the first-generation immigrants, 0.5% were diagnosed with gout, i.e. 0.8% among men and 0.3% among women, while among second-generation immigrants, 0.3% were diagnosed with gout. In general, the incidence of gout tended to be lower among both first- and second-generation immigrants than Swedish born, higher among lower educated and lower among higher educated, higher among residents in the larger cities and lower in northern Sweden and higher among individuals with co-morbidities, especially cardio-vascular co-morbidities.

**Table 1:** Population and number of cases of events (with percentages) in first generation and second generation

	First generation				Second generation			
	Population		Gout events		Population		Gout events	
	No.	%	No.	%	No.	%	No.	%
Total population	6,449,369		32,956		6,874,682		18,212	
Gender								
Males	3,051,102	47.3	22,919	69.5	3,508,632	51.0	14,511	79.7
Females	3,398,267	52.7	10,037	30.5	3,366,050	49.0	3701	20.3
Immigrant status^a^								
Sweden	5,306,288	82.3	28,900	87.7	5,666,670	82.4	16,287	89.4
Other countries	1,143,081	17.7	4056	12.3	1,208,012	17.6	1925	10.6
Birth year								
−1909	79,034	1.2	328	1.0				
1910–19	349,829	5.4	3615	11.0				
1920–29	628,432	9.7	8270	25.1				
1930–39	733,408	11.4	7534	22.9	447,284	6.5	4246	23.3
1940–49	1,093,953	17.0	6495	19.7	1,039,756	15.1	6119	33.6
1950–59	1,027,898	15.9	3702	11.2	1,035,473	15.1	3812	20.9
1960–69	1,101,200	17.1	2129	6.5	1,153,201	16.8	2370	13.0
1970–	1,435,615	22.3	883	2.7	3,198,968	46.5	1665	9.1
Educational level								
≤9	2,015,602	31.3	15,352	46.6	1,772,765	25.8	5894	32.4
10–12	1,628,711	25.3	8570	26.0	1,476,803	21.5	5791	31.8
>12	2,805,056	43.5	9034	27.4	3,625,114	52.7	6527	35.8
Region of residence								
Large cities	2,068,774	32.1	13,444	40.8	2,046,576	29.8	7463	41.0
Southern Sweden	2,696,245	41.8	14,039	42.6	2,653,219	38.6	7619	41.8
Northern Sweden	1,684,350	26.1	5473	16.6	2,174,887	31.6	3130	17.2
Marital status								
Married	4,758,618	73.8	23,425	71.1	4,719,785	68.7	10,860	59.6
Unmarried	1,690,751	26.2	9531	28.9	2,154,897	31.3	7352	40.4
Neighbourhood deprivation								
Low	891,126	13.8	4420	13.4	939,288	13.7	2668	14.6
Middle	3,043,079	47.2	16,065	48.7	2,977,688	43.3	8644	47.5
High	722,008	11.2	3720	11.3	684,085	10.0	1979	10.9
Unknown	1,793,156	27.8	8751	26.6	2,273,621	33.1	4921	27.0
Hospital diagnosis of COPD								
No	6,160,669	95.5	28,777	87.3	6,598,004	96.0	16,394	90.0
Yes	288,700	4.5	4179	12.7	276,678	4.0	1818	10.0
Hospital diagnosis of obesity								
No	6,364,017	98.7	31,844	96.6	6,768,237	98.5	17,216	94.5
Yes	85,352	1.3	1112	3.4	106,445	1.5	996	5.5
Hospital diagnosis of CHD								
No	5,916,045	91.7	22,145	67.2	6,656,575	96.8	14,577	80.0
Yes	533,324	8.3	10,811	32.8	218,107	3.2	3635	20.0
Hospital diagnosis of diabetes								
No	6,099,921	94.6	26,181	79.4	6,656,003	96.8	14,950	82.1
Yes	349,448	5.4	6775	20.6	218,679	3.2	3262	17.9
Hospital diagnosis of alcoholism and related disorders								
No	6,313,123	97.9	31,197	94.7	6,689,528	97.3	16,531	90.8
Yes	136,246	2.1	1759	5.3	185,154	2.7	1681	9.2
Hospital diagnosis of stroke								
No	6,077,938	94.2	27,275	82.8	6,741,870	98.1	16,462	90.4
Yes	371,431	5.8	5681	17.2	132,812	1.9	1750	9.6
Hospital diagnosis of hypertension								
No	5,689,298	88.2	18,884	57.3	6,422,170	93.4	11,053	60.7
Yes	760,071	11.8	14,072	42.7	452,512	6.6	7159	39.3
Hospital diagnosis of heart failure								
No	6,126,173	95.0	22,043	66.9	6,797,635	98.9	15,298	84.0
Yes	323,196	5.0	10,913	33.1	77,047	1.1	2914	16.0

All differences between the population and patients diagnosed with gout were statistically significant (*p* < 0.001)

^a^Immigrant status in second generation was based on parental birth country

Table 2 presents risk of gout in first-generation male immigrants compared to their Swedish-born counterparts. After adjustment for age, region of residence in Sweden, educational level, marital status and neighbourhood deprivation (model 3) and compared to Swedish-born men, the risk of gout was higher among male immigrants with origin from Austria; Poland; Russia; and African and Asian continents, especially Iraq. In contrast, compared to Swedish-born men, the risk of gout was lower in men originating from Denmark; Norway; Southern Europe, especially Greece and Spain; and Latin America, especially Chile. After additional adjustment for co-morbidities (model 4), the estimates were somewhat attenuated but mostly with marginal changes.

**Table 2:** The risk of gout in first-generation male immigrants

Country/region of origin	Model 1	Model 2	Model 3	Model 4
	HR (95% CI)	HR (95% CI)	HR (95% CI)	HR (95% CI)
Sweden	1 (ref)	1 (ref)	1 (ref)	1 (ref)
*Nordic countries*	*0.93* (*0.87*–*0.99*)	*0.92* (*0.86*–*0.98*)	0.94 (0.88–1.00)	*0.87* (*0.82*–*0.93*)
Denmark	*0.75* (*0.64*–*0.88*)	*0.74* (*0.64*–*0.87*)	*0.75* (*0.64*–*0.88*)	*0.76* (*0.65*–*0.89*)
Finland	1.05 (0.90–1.13)	1.03 (0.95–1.10)	1.06 (0.98–1.14)	0.94 (0.87–1.01)
Norway	*0.70* (*0.58*–*0.85*)	*0.71* (*0.59*–*0.85*)	*0.72* (*0.60*–*0.87*)	*0.74* (*0.62*–*0.90*)
*Southern Europe*	*0.65* (*0.55*–*0.78*)	*0.65* (*0.54*–*0.77*)	*0.67* (*0.56*–*0.80*)	*0.74* (*0.62*–*0.88*)
France	0.72 (0.42–1.24)	0.74 (0.43–1.28)	0.77 (0.45–1.33)	0.81 (0.47–1.40)
Greece	*0.46* (*0.33*–*0.63*)	*0.45* (*0.32*–*0.62*)	*0.47* (*0.34*–*0.65*)	*0.54* (*0.39*–*0.76*)
Italy	0.87 (0.65–1.15)	0.86 (0.65–1.14)	0.89 (0.67–1.18)	0.91 (0.68–1.20)
Spain	*0.50* (*0.29*–*0.87*)	*0.49* (*0.29*–*0.85*)	*0.51* (*0.30*–*0.89*)	*0.57* (*0.33*–*0.98*)
Other Southern Europe	1.03 (0.64–1.66)	1.02 (0.63–1.64)	1.03 (0.64–1.66)	1.16 (0.72–1.87)
*Western Europe*	1.08 (0.97–1.21)	1.11 (0.99–1.24)	*1.13 (1.01–1.26)*	*1.14 (1.02–1.28)*
The Netherlands	0.60 (0.35–1.03)	0.62 (0.36–1.06)	0.63 (0.37–1.09)	0.66 (0.38–1.14)
UK and Ireland	1.03 (0.80–1.33)	1.06 (0.82–1.37)	1.10 (0.85–1.42)	1.24 (0.96–1.61)
Germany	1.11 (0.96–1.29)	1.14 (0.99–1.32)	1.16 (1.00–1.34)	1.13 (0.98–1.31)
Austria	*1.41* (*1.08*–*1.85*)	*1.45* (*1.10*–*1.90*)	*1.48* (*1.13*–*1.95*)	*1.42* (*1.08*–*1.87*)
Other Western Europe	0.79 (0.40–1.40)	0.82 (0.47–1.45)	0.85 (0.48–1.50)	0.89 (0.51–1.57)
*Eastern Europe*	1.08 (0.97–1.21)	1.09 (0.98–1.22)	1.11 (1.00–1.25)	1.03 (0.92–1.15)
Bosnia	0.96 (0.72–1.29)	1.02 (0.76–1.37)	1.11 (0.83–1.49)	0.86 (0.64–1.16)
Yugoslavia	1.09 (0.95–1.25)	1.08 (0.94–1.24)	1.09 (0.95–1.26)	1.03 (0.90–1.19)
Croatia	1.46 (0.95–2.25)	1.46 (0.95–2.24)	1.45 (0.94–2.22)	1.47 (0.96–2.26)
Romania	*1.42* (*1.02*–*1.99*)	*1.46* (*1.05*–*2.05*)	*1.48* (*1.06*–*2.07*)	1.39 (0.99–1.95)
*Baltic countries*	0.83 (0.63–1.08)	0.85 (0.65–1.11)	0.86 (0.66–1.13)	0.83 (0.63–1.09)
Estonia	0.78 (0.58–1.06)	0.80 (0.59–1.08)	0.81 (0.60–1.10)	0.78 (0.58–1.06)
Latvia	1.07 (0.60–1.94)	1.12 (0.62–2.02)	1.14 (0.63–2.06)	1.08 (0.60–1.95)
*Central Europe*	*1.25* (*1.09*–*1.43*)	*1.28* (*1.12*–*1.46*)	*1.29* (*1.13*–*1.47*)	*1.18* (*1.04*–*1.35*)
Poland	*1.38* (*1.14*–*1.66*)	*1.41* (*1.17*–*1.70*)	*1.42* (*1.18*–*1.72*)	*1.31* (*1.08*–*1.58*)
Other Central Europe	1.01 (0.72–1.42)	1.05 (0.75–1.48)	1.06 (0.75–1.49)	1.01 (0.72–1.42)
Hungary	1.22 (0.98–1.51)	1.24 (1.00–1.54)	1.24 (1.00–1.55)	1.12 (0.90–1.40)
*Africa*	*1.33* (*1.12*–*1.59*)	*1.34* (*1.12*–*1.59*)	*1.40* (*1.17*–*1.67*)	*1.36* (*1.14*–*1.63*)
*Northern America*	0.80 (0.60–1.07)	0.83 (0.62–1.11)	0.86 (0.64–1.14)	0.91 (0.68–1.22)
*Latin America*	*0.59* (*0.46*–*0.76*)	*0.59* (*0.45*–*0.76*)	*0.61* (*0.47*–*0.78*)	*0.66* (*0.51*–*0.85*)
Chile	*0.48* (*0.33*–*0.68*)	*0.47* (*0.33*–*0.67*)	*0.48* (*0.34*–*0.69*)	*0.52* (*0.36*–*0.74*)
South America	0.78 (0.55–1.12)	0.79 (0.55–1.13)	0.82 (0.57–1.17)	0.91 (0.64–1.31)
*Asia*	*1.28* (*1.18*–*1.40*)	*1.30* (*1.19*–*1.42*)	*1.35* (*1.24*–*1.47*)	*1.28* (*1.17*–*1.39*)
Turkey	0.91 (0.73–1.13)	0.89 (0.71–1.11)	0.93 (0.75–1.16)	0.89 (0.71–1.11)
Lebanon	1.03 (0.75–1.43)	1.01 (0.73–1.39)	1.04 (0.75–1.44)	0.99 (0.72–1.37)
Iran	1.10 (0.91–1.33)	1.15 (0.95–1.39)	1.17 (0.97–1.42)	1.20 (1.00–1.45)
Iraq	*2.00* (*1.69*–*2.37*)	*2.10* (*1.77*–*2.48*)	*2.22* (*1.87*–*2.63*)	*1.82* (*1.54*–*2.16*)
Other Asia countries	*1.37* (*1.18*–*1.59*)	*1.39* (*1.20*–*1.61*)	*1.44* (*1.24*–*1.67*)	*1.37* (*1.18*–*1.59*)
*Russia*	*1.87* (*1.39*–*2.50*)	*1.91* (*1.43*–*2.56*)	*1.95* (*1.46*–*2.62*)	*1.69* (*1.26*–*2.27*)

﻿Regions (also including separately listed countries) marked by italicsHR (95% CI): Hazard ratios with 95% confidence intervalStatistically significant HRs marked by italics﻿Model 1: adjusted for age and region of residence in Sweden; model 2: adjusted for age, region of residence in Sweden, educational level and marital status; model 3: model 2 + neighbourhood deprivation; model 4: model 3 + co-morbidities

Table 3 presents risk of gout in first-generation female immigrants compared to Swedish-born women. After adjustment for age, region of residence in Sweden, educational level, marital status and neighbourhood deprivation (model 3), the risk of gout was higher among immigrant women from Austria; Romania; Central Europe, especially Poland and Hungary; Africa and Asia, especially Turkey and Iraq; while a lower risk of gout was observed for immigrant women with origin not only from Southern Europe but also from North America compared to Swedish-born women. Additional adjustment for co-morbidities (model 4) attenuated the estimates, and these were no longer significant for women from Romania and Turkey and were with borderline significance among Polish women. When performing a sensitive analysis excluding immigrants who moved to Sweden within 5 years of follow-up, the estimates were very similar (Supplementary Tables 2 and 3).

**Table 3:** The risk of gout in first-generation female immigrants

Country/region of origin	Model 1	Model 2	Model 3	Model 4
	HR (95% CI)	HR (95% CI)	HR (95% CI)	HR (95% CI)
Sweden	1 (ref)	1 (ref)	1 (ref)	1 (ref)
*Nordic countries*	1.05 (0.97–1.15)	1.01 (0.93–1.10)	1.03 (0.94–1.12)	0.94 (0.87–1.03)
Denmark	0.96 (0.76–1.21)	0.93 (0.74–1.17)	0.93 (0.74–1.17)	0.96 (0.76–1.21)
Finland	*1.14* (*1.03*–*1.26*)	1.09 (0.98–1.20)	*1.11 (1.01–1.23)*	0.98 (0.89–1.09)
Norway	0.88 (0.71–1.09)	0.85 (0.69–1.06)	0.86 (0.69–1.06)	0.82 (0.66–1.02)
*Southern Europe*	*0.53* (*0.35*–*0.81*)	*0.50* (*0.33*–*0.76*)	*0.52* (*0.52*–*0.78*)	*0.61* (*0.40*–*0.93*)
*Western Europe*	1.05 (0.88–1.25)	1.09 (0.91–1.30)	1.11 (0.93–1.33)	1.09 (0.91–1.30)
The Netherlands	0.71 (0.27–1.90)	0.75 (0.28–2.00)	0.78 (0.29–2.07)	0.84 (0.32–2.24)
Germany	1.05 (0.85–1.29)	1.09 (0.88–1.34)	1.11 (0.90–1.36)	1.04 (0.84–1.28)
Austria	*1.68* (*1.06*–*2.67*)	*1.74* (*1.10*–*2.77*)	*1.77* (*1.12*–*2.82*)	*1.70* (*1.07*–*2.70*)
*Eastern Europe*	*1.26* (*1.02*–*1.57*)	1.19 (0.96–1.48)	1.21 (0.97–1.50)	1.10 (0.89–1.36)
Bosnia	1.33 (0.77–2.30)	1.34 (0.78–2.32)	1.47 (0.85–2.54)	1.17 (0.68–2.02)
Yugoslavia	1.14 (0.86–1.50)	1.04 (0.79–1.38)	1.04 (0.79–1.38)	0.96 (0.73–1.27)
Romania	*1.86* (*1.06*–*3.28*)	*2.00* (*1.14*–*3.53*)	*2.02* (*1.14*–*3.55*)	1.72 (0.98–3.04)
*Baltic countries*	0.87 (0.61–1.24)	0.94 (0.66–1.34)	0.96 (0.67–1.37)	0.89 (0.63–1.27)
Estonia	0.73 (0.48–1.11)	0.78 (0.51–1.18)	0.80 (0.52–1.21)	0.75 (0.49–1.13)
*Central Europe*	*1.55* (*1.27*–*1.88*)	*1.65* (*1.36*–*2.01*)	*1.65* (*1.36*–*2.01*)	*1.47* (*1.21*–*1.79*)
Poland	*1.42* (*1.08*–*1.87*)	*1.50* (*1.14*–*1.98*)	*1.50* (*1.14*–*1.98*)	*1.34* (*1.01*–*1.76*)
Other Central Europe	1.00 (0.57–1.76)	1.09 (0.62–1.92)	1.10 (0.62–1.94)	1.06 (0.60–1.86)
Hungary	*2.20* (*1.61*–*3.01*)	*2.34* (*1.71*–*3.21*)	*2.34* (*1.71*–*3.21*)	*1.98* (*1.45*–*2.71*)
*Africa*	*2.32* (*1.57*–*3.44*)	*2.15* (*1.45*–*3.19*)	*2.27* (*1.53*–*3.36*)	*2.23* (*1.50*–*3.31*)
*Northern America*	*0.36* (*0.18*–*0.72*)	*0.37* (*0.19*–*0.74*)	*0.38* (*0.19*–*0.76*)	*0.41* (*0.21*–*0.83*)
*Latin America*	0.82 (0.52–1.29)	0.80 (0.51–1.25)	0.82 (0.52–1.28)	0.83 (0.53–1.30)
Chile	0.90 (0.51–1.59)	0.85 (0.48–1.50)	0.87 (0.49–1.53)	0.84 (0.47–1.47)
*Asia*	*1.40* (*1.16*–*1.69*)	*1.31* (*1.08*–*1.58*)	*1.35* (*1.11*–*1.63*)	1.20 (0.99–1.45)
Turkey	*1.66* (*1.16*–*2.37*)	1.41 (0.99–2.03)	*1.46* (*1.02*–*2.09*)	1.16 (0.81–1.66)
Iraq	*2.19* (*1.41*–*3.41*)	*2.12* (*1.36*–*3.30*)	*2.28* (*1.46*–*3.55*)	*1.76* (*1.13*–*2.74*)
Other Asia countries	*1.36* (*1.01*–*1.84*)	1.29 (0.96–1.75)	1.33 (0.99–1.80)	1.30 (0.96–1.75)
*Russia*	0.87 (0.49–1.53)	0.93 (0.53–1.63)	0.94 (0.53–1.66)	0.84 (0.48–1.49)

Regions (also including separately listed countries) marked by italicsHR (95% CI): Hazard ratios with 95% confidence intervalStatistically significant HRs marked by italics﻿ Model 1: adjusted for age and region of residence in Sweden; model 2: adjusted for age, region of residence in Sweden, educational level and marital status; model 3: model 2 + neighbourhood deprivation; model 4: model 3 + co-morbidities

Tables 4 and 5 present the risk of gout in second-generation male and female immigrants, respectively, compared to their Swedish-born counterparts. After adjusting for age, region of residence, educational level, marital status and neighbourhood SES (model 3), the risk of gout was higher among male immigrants with origin from the Nordic countries, especially Finland, and from Central Europe, especially Poland and in immigrants from the former Yugoslavia. In contrast, compared to Swedish-born men, the risk of gout was lower in men originating from Greece. Furthermore, second-generation immigrant women from the Nordic countries had lower risk for gout compared to their Swedish-born counterparts. With additional adjustment for co-morbidities (model 4), the estimates were somewhat attenuated.

**Table 4:** The risk of gout in second-generation male immigrants

Country/region of origin	Model 1	Model 2	Model 3	Model 4
	HR (95% CI)	HR (95% CI)	HR (95% CI)	HR (95% CI)
Sweden	1 (ref)			
*Nordic countries*	*1.23* (*1.15*–*1.32*)	*1.20* (*1.23*–*1.29*)	*1.18* (*1.11*–*1.26*)	*1.17* (*1.10*–*1.25*)
Denmark	1.08 (0.92–1.27)	1.06 (0.90–1.25)	1.07 (0.91–1.25)	1.12 (0.95–1.31)
Finland	*1.34* (*1.23*–*1.45*)	*1.29* (*1.19*–*1.41*)	*1.26* (*1.16*–*1.37*)	*1.23* (*1.13*–*1.33*)
Norway	1.14 (0.99–1.31)	1.13 (0.98–1.30)	1.12 (0.97–1.29)	1.11 (0.96–1.28)
*Southern Europe*	0.93 (0.70–1.23)	0.92 (0.70–1.22)	0.89 (0.67–1.18)	0.97 (0.73–1.28)
Greece	*0.47* (*0.24*–*0.95*)	*0.46* (*0.23*–*0.93*)	*0.44* (*0.22*–*0.89*)	0.50 (0.25–1.00)
Italy	1.34 (0.89–2.01)	1.32 (0.88–1.99)	1.28 (0.85–1.93)	1.39 (0.92–2.09)
Spain	1.45 (0.80–2.62)	1.43 (0.79–2.59)	1.38 (0.77–2.50)	1.45 (0.80–2.62)
*Western Europe*	0.96 (0.82–1.12)	0.98 (0.84–1.14)	0.96 (0.82–1.12)	1.00 (0.86–1.17)
The Netherlands	0.83 (0.41–1.66)	0.84 (0.42–1.68)	0.83 (0.41–1.65)	0.89 (0.45–1.78)
UK and Ireland	0.63 (0.36–1.12)	0.65 (0.37–1.15)	0.63 (0.36–1.12)	0.65 (0.37–1.15)
Germany	1.01 (0.84–1.21)	1.02 (0.85–1.22)	1.01 (0.84–1.21)	1.05 (0.88–1.26)
Austria	1.28 (0.85–1.93)	1.31 (0.87–1.97)	1.28 (0.85–1.93)	1.31 (0.87–1.98)
*Eastern Europe*	1.21 (0.97–1.52)	1.23 (0.98–1.54)	1.21 (0.97–1.51)	1.18 (0.95–1.48)
Yugoslavia	*1.31* (*1.02*–*1.70*)	*1.30* (*1.01*–*1.67*)	1*.30* (*1.00*–*1.67*)	1.28 (0.99–1.65)
Romania	1.47 (0.73–2.93)	1.52 (0.76–3.03)	1.49 (0.75–2.99)	1.42 (0.71–2.85)
*Baltic countries*	1.17 (0.93–1.47)	1.20 (0.96–1.51)	1.17 (0.93–1.47)	1.20 (0.96–1.51)
Estonia	1.15 (0.90–1.48)	1.18 (0.92–1.51)	1.15 (0.90–1.48)	1.20 (0.93–1.53)
Latvia	1.29 (0.73–2.27)	1.34 (0.76–2.36)	1.31 (0.75–2.31)	1.25 (0.71–2.20)
*Central Europe*	*1.37* (*1.11*–*1.69*)	*1.40* (*1.13*–*1.72*)	*1.39* (*1.13*–*1.72*)	*1.36* (*1.10*–*1.68*)
Poland	*1.46* (*1.09*–*1.96*)	*1.52* (*1.13*–*2.03*)	*1.51* (*1.13*–*2.02*)	*1.45* (*1.08*–*1.94*)
Other Central Europe	1.20 (0.72–1.98)	1.22 (0.74–2.03)	1.21 (0.73–2.01)	1.24 (0.75–2.05)
Hungary	1.37 (0.94–1.98)	1.36 (0.94–1.97)	1.36 (0.94–1.97)	1.34 (0.92–1.94)
*North America*	0.99 (0.75–1.29)	1.01 (0.77–1.32)	1.00 (0.76–1.30)	1.02 (0.78–1.34)
*Latin America*	0.70 (0.41–1.18)	0.70 (0.42–1.19)	0.68 (0.40–1.15)	0.66 (0.39–1.11)
South America	1.11 (0.58–2.14)	1.15 (0.60–2.21)	1.12 (0.58–2.15)	1.11 (0.58–2.13)
*Asia*	1.02 (0.81–1.27)	1.04 (0.83–1.30)	1.00 (0.80–1.25)	0.95 (0.76–1.19)
Turkey	0.95 (0.63–1.44)	0.93 (0.61–1.40)	0.89 (0.59–1.35)	0.89 (0.59–1.34)
Iran	1.12 (0.65–1.93)	1.20 (0.69–2.06)	1.17 (0.68–2.01)	1.07 (0.62–1.84)
Iraq	1.34 (0.76–2.36)	1.45 (0.82–2.56)	1.34 (0.76–2.36)	1.15 (0.65–2.03)
Other Asian countries	1.10 (0.76–1.59)	1.14 (0.79–1.66)	1.10 (0.76–1.60)	1.07 (0.74–1.55)
*Russia*	0.85 (0.53–1.37)	0.86 (0.53–1.38)	0.85 (0.53–1.36)	0.82 (0.51–1.32)

Regions (also including separately listed countries) marked by italicsHR (95% CI): Hazard ratios with 95% confidence intervalStatistically significant HRs marked by italics Model 1: adjusted for age and region of residence in Sweden; model 2: adjusted for age, region of residence in Sweden, educational level and marital status; model 3: model 2 + neighbourhood deprivation; model 4: model 3 + co-morbidities

**Table 5:** The risk of gout in second-generation female immigrants

Country/region of origin	Model 1	Model 2	Model 3	Model 4
	HR (95% CI)	HR (95% CI)	HR (95% CI)	HR (95% CI)
Sweden	1 (ref)	1 (ref)	1 (ref)	1 (ref)
*Nordic countries*	0.88 (0.76–1.03)	*0.84* (*0.72*–*0.98*)	*0.82* (*0.70*–*0.96*)	*0.83* (*0.71*–*0.97*)
Denmark	0.74 (0.50–1.10)	0.69 (0.47–1.03)	0.69 (0.47–1.02)	0.74 (0.50–1.09)
Finland	0.94 (0.77–1.14)	0.90 (0.74–1.09)	0.87 (0.72–1.06)	0.85 (0.70–1.04)
Norway	0.89 (0.65–1.21)	0.85 (0.62–1.17)	0.85 (0.62–1.16)	0.87 (0.64–1.20)
*Southern Europe*	0.79 (0.42–1.47)	0.70 (0.38–1.31)	0.67 (0.36–1.25)	0.77 (0.41–1.44)
Greece	0.75 (0.24–2.33)	0.62 (0.20–1.94)	0.59 (0.19–1.84)	0.72 (0.23–2.23)
Italy	0.49 (0.12–1.98)	0.45 (0.11–1.81)	0.43 (0.11–1.74)	0.52 (0.13–2.09)
Spain	1.17 (0.29–4.70)	1.08 (0.27–4.31)	1.04 (0.26–4.16)	1.04 (0.26–4.17)
*Western Europe*	0.70 (0.49–1.01)	0.70 (0.49–1.01)	*0.69* (*0.48*–*0.99*)	0.76 (0.53–1.09)
The Netherlands	0.87 (0.22–3.48)	0.84 (0.21–3.36)	0.82 (0.20–3.27)	0.96 (0.24–3.83)
UK and Ireland	1.06 (0.44–2.55)	1.01 (0.42–2.42)	0.97 (0.40–2.34)	1.06 (0.44–2.55)
Germany	0.64 (0.40–1.02)	0.65 (0.41–1.03)	0.64 (0.40–1.01)	0.70 (0.44–1.11)
Austria	0.68 (0.22–2.09)	0.69 (0.22–2.14)	0.68 (0.22–2.10)	0.74 (0.24–2.30)
*Eastern Europe*	1.11 (0.69–1.79)	1.03 (0.64–1.67)	0.99 (0.62–1.61)	1.01 (0.62–1.63)
Yugoslavia	0.94 (0.50–1.75)	0.86 (0.46–1.60)	0.84 (0.45–1.56)	0.88 (0.47–1.63)
Romania	2.30 (0.74–7.13)	2.28 (0.74–7.08)	2.21 (0.71–6.85)	2.07 (0.67–6.42)
*Baltic countries*	1.15 (0.73–1.80)	1.19 (0.76–1.86)	1.16 (0.74–1.82)	1.22 (0.77–1.91)
Estonia	1.06 (0.64–1.77)	1.09 (0.66–1.82)	1.07 (0.64–1.78)	1.13 (0.68–1.87)
Latvia	1.62 (0.61–4.33)	1.77 (0.66–4.71)	1.72 (0.64–4.58)	1.78 (0.67–4.75)
*Central Europe*	1.28 (0.82–1.99)	1.27 (0.82–1.97)	1.26 (0.81–1.96)	1.25 (0.81–1.95)
Poland	1.20 (0.62–2.30)	1.19 (0.62–2.29)	1.18 (0.61–2.26)	1.12 (0.58–2.16)
Other Central Europe	1.28 (0.48–3.42)	1.28 (0.48–3.42)	1.27 (0.48–3.38)	1.33 (0.50–3.54)
Hungary	1.42 (0.67–2.97)	1.41 (0.67–2.96)	1.40 (0.67–2.95)	1.45 (0.69–3.05)
*North America*	0.75 (0.41–1.35)	0.75 (0.42–1.36)	0.74 (0.41–1.34)	0.80 (0.44–1.44)
*Latin America*	1.32 (0.59–2.94)	1.17 (0.52–2.61)	1.12 (0.50–2.50)	1.05 (0.47–2.35)
South America	1.62 (0.52–5.04)	1.47 (0.47–4.57)	1.42 (0.46–4.40)	1.42 (0.46–4.41)
*Asia*	1.42 (0.95–2.11)	1.25 (0.83–1.86)	1.18 (0.79–1.77)	1.10 (0.74–1.65)
Turkey	*2.04* (*1.13*–*3.70*)	1.74 (0.96–3.16)	1.66 (0.91–3.01)	1.61 (0.89–2.92)
Iran	0.40 (0.06–2.79)	0.37 (0.05–2.62)	0.36 (0.05–2.52)	0.32 (0.05–2.24)
Iraq	1.38 (0.44–4.30)	1.18 (0.38–3.67)	1.09 (0.35–3.41)	0.94 (0.30–2.94)
Other Asian countries	1.70 (0.91–3.17)	1.54 (0.83–2.88)	1.48 (0.79–2.76)	1.42 (0.76–2.66)
*Russia*	0.92 (0.38–2.21)	0.93 (0.39–2.23)	0.91 (0.38–2.19)	0.87 (0.36–2.09)

Regions (also including separately listed countries) marked by italicsHR (95% CI): Hazard ratios with 95% confidence intervalStatistically significant HRs marked by italics Model 1: adjusted for age and region of residence in Sweden; model 2: adjusted for age, region of residence in Sweden, educational level and marital status; model 3: model 2 + neighbourhood deprivation; model 4: model 3 + co-morbidities

## Discussion

We explored the risk of being diagnosed with gout in first- and second-generation immigrant men and women than their Swedish-born counterparts, with a total prevalence of 0.5%. The most prominent differences in rates of gout were observed between first-generation immigrants and in Swedish-born people. Risk of being diagnosed with gout was higher not only among first-generation immigrants from several European countries but also among immigrants from Africa and some Asian countries, while the risk of being diagnosed with gout was lower among first-generation immigrants in some Southern European countries and among second-generation immigrant men from Greece. The differences in gout rates between the second-generation immigrant men and their Swedish-born counterparts were less apparent, while almost no difference was noted among women. The latter, however, may have been due to a low number of events.

When looking at the risk of gout among immigrants from European countries and the estimated prevalence in these countries, some interesting differences could be noted. Prevalence of gout is reported to be highest in Greece, with slightly lower figures from the UK, the Netherlands and Spain, while the lowest prevalence is reported from Portugal and the Czech Republic, with also low figures from France and Italy [16]. We found lower estimates for immigrants from Southern Europe, for men among first-generation immigrants from Greece and Spain, and among second-generation immigrants from Greece, in contrast to the reported prevalence figures from these countries. Low prevalence of gout in the world is reported from Asian countries, including the Middle East, African countries and at least from one country, i.e. Guatemala, in Latin America [16]. In contrast to these findings, we found higher gout risk among first-generation immigrants from Middle East countries and Africa.

We have no certain explanation for the disagreement between our study and earlier prevalence figures, e.g. the high gout prevalence in Greece and the low gout risk among Greek immigrants we found. However, emigrants from a specific country or region may differ from those staying in their country of origin, i.e. migrants often tend to be healthier (the “healthy migrant effect”), even if many immigrants retain many of their dietary habits. The low estimates in some groups could possibly be related to the healthy migrant effect, e.g. men from Denmark and Norway and women from Northern America. For second-generation immigrants, an adaptation to the mainstream dietary culture could be expected, which could explain the regression towards the reference population, i.e. individuals with indigenous parents. Besides, comparing prevalence figures between studies of diagnosed gout in published papers is difficult, as there is a large variation in the prevalence of gout depending on different factors, including the used definition of gout, the sampling methods, the studied age groups and the sex distribution, with a higher prevalence in men [9, 21]. For instance, we emphasize that the gout prevalence of 0.5% in the present study is in accordance with the prevalence figure worldwide found in a review, which is 0.6% [9]. The prevalence in the present study was based on a hospital diagnosis of gout. A similar prevalence was found in an earlier Swedish study on reported diagnosis of gout from all care branches, i.e. hospital in-care, specialized open care and primary care [22]. However, in that study, the prevalence was 1.4% when including all individuals ever reported with a gout diagnosis including from primary health care being alive at the end of the study period [22], i.e. around the same prevalence as in the UK and Germany [15], and on the same level as in North America and other countries in Western Europe [16].

Regarding the situation among different immigrant groups, the higher gout prevalence in different ethnic groups, including Filipinos and African Americans, has been attributed to the high gout prevalence in North America [23]. The higher risk of hyperuricemia and gout as described among Filipinos in the USA is ascribed to the shift from a low-purine diet to a high-purine Western diet, and the Filipino group seems to be especially vulnerable when adapting a high-purine diet [23].

When trying to understand the risk pattern of gout among the immigrant groups found in our study, the pattern of risk factors for gout is of importance. Gout is strongly related to lifestyle and dietary habits [24], including alcohol intake [12]. Trends in lifestyle factors, such as the increased alcohol intake [25], and change in dietary habits, including higher intake of sweetened soft drinks [21], contribute to increased obesity and the metabolic syndrome in the general population. High alcohol intake, especially of beer, is also a factor of importance for hyperuricemia and gout, while intake of wine rather seems to be protective [25]. According to the OECD statistics, alcohol intake per capita was highest in Estonia and Austria in 2012, with Hungary, Russia and Germany ranked on the seventh to ninth places, and with a very low alcohol intake in Turkey [26]. However, pattern of intake of the different alcohol beverages differ, with a high intake of beer in inhabitants not only in Austria, Germany, Finland, Poland, Hungary, Russia and the Netherlands but also in Spain; a high intake of spirits in Russia, Hungary and Poland; and a high intake of wine and relatively lower of other alcohol beverages in France, Greece, Italy and Chile. Immigrants probably bring drinking habits from their country of origin, which is why the alcohol intake patterns of home countries is of importance. Thus, high intake of beer or spirits could partly explain the higher risk of gout among immigrants from Finland, Austria, Poland, Hungary and Russia.

The increased prevalence of gout and hyperuricemia in both Western and third-world societies has been linked not only to urbanization, western lifestyle and immigration to western countries [27] but also to higher rates of hypertension [28]. The increased incidence and prevalence of gout worldwide tails the obesity epidemic [11]. Even if gout has been regarded as “the disease of kings” and as such associated to wealth and good living [29], it is nowadays more linked to lower socio-economic status [30], which is also in line with our findings. The risk of overweight and obesity, especially abdominal obesity, has been shown to be higher in many immigrant groups of non-European origin [31]. Among non-Western immigrants, Middle East women in general have a higher risk of abdominal obesity [32] and diabetes [33]. When looking at dietary patterns among immigrants, also including second-generation immigrants, a pattern with more sugar-rich food and beverages and more fat-rich food is seen [31]. In general, it seems that dietary habits among non-Western immigrant groups in Europe are likely to become less healthy [34]. Dietary habits among second-generation immigrants also tend to approach those found in age- and sex-matched individuals in the indigenous population [35]. In contrast, the prevalence of hypertension is lower among immigrants of non-European origin in Sweden [36], i.e. one important factor that could explain the lower risk of gout among some groups. As hyperuricemia and gout also is associated with CVD and CHD, patterns of these among different countries are of importance. According to recent statistics from the OECD, the CHD mortality is higher in Finland, the Baltic countries, Eastern Europe, Hungary and Turkey than in Sweden; while a lower CHD mortality is reported from the Nordic countries with the exception of Finland, Southern Europe and Chile [37].

When looking at the specific immigrant groups and the risk of gout, among Turkish born women an increased risk of abdominal obesity and the metabolic syndrome [38] and also of diabetes [39], has been reported. Besides, among Iraqi immigrants, an increased risk of pre-diabetes and diabetes has been reported [40]. A higher risk of abdominal obesity is also found among immigrants, especially in women, from Eastern Europe, in that study also, including immigrants from Poland, Hungary and Russia [32], and a higher body mass index (BMI) among Polish men [41]. The lower estimates in some of these groups when adjusting for co-morbidities could support that obesity may partly explain the excess risk. Otherwise, the estimates were only marginally changed when adjusting for co-morbidities. As regards the lower risk of gout among South Europeans, adherence to Mediterranean diet is associated with a lower risk of hyperuricemia [42].

There are some limitations in this study. As we divided into many different immigrant groups, there is a risk of mass significance when using the *p* level < 0.05. Thus, the results should be interpreted with caution owing to the multiple testing. The statistical power to detect significant results also differed between the immigrant groups owing to varying sample size, and the power was lower among women, especially second-generation women. We used hospital diagnoses to identify individuals with gout, and it is possible that other groups would have been identified if we had access to diagnoses in other care forms, especially primary care. As diagnoses were taken from the National Patient Register, they are clinically based and we cannot check for the criteria for gout diagnosis being used. Furthermore, we had no access to prescription of allopurinol, but when this was actual, the diagnosis was probably set. We adjusted for co-morbidities which can be discussed as the association between gout and co-morbidities may go in both directions, i.e. may be a risk factor for gout or a consequence of gout. However, results when adjusted for co-morbidities were similar in most cases. Despite the limitations, this is one of rare studies in Europe providing insight in differences in gout among different immigrant groups. A strength of the study is that we used neighbourhood SES that is a proxy for lifestyle factors [43], and it has been shown to be associated with, for example smoking [44, 45].

In conclusion, the results of this study indicate significant differences in risks of gout between immigrants and Swedish-born groups, which are more prominent between first-generation immigrant men and women and their Swedish counterparts. As gout is a risk factor for cardio-vascular diseases, including an increased mortality risk [10], identifying patients with hyperuricemia and gout is one way to be able to prevent these diseases. The differences observed may be related to differences between immigrants and Swedish born in the interplay between genetics and environment, risk factors for gout or provision of medical care. As gout causes suffering for the individuals and increased cost for society, measures in preventing gout, especially by healthier lifestyle habits, could be an effective way to entangle the problem. As the risk of gout is increased among some immigrant groups, it is important to draw attention to this, to reassure equity in health in the population. A healthier lifestyle in the whole population is desirable, but to reach this goal, interventions might need to be tailored for specific groups.

## Electronic supplementary material

ESM 1 (DOCX 19.3 kb)

ESM 2 (DOCX 13.8 kb)

ESM 3 (DOCX 13.3 kb)
