# Supplementary material for: Gout in immigrant groups: a cohort study in Sweden
Source: Clin Rheumatol. 2017 Jan 13;36(5):1091–102. doi: 10.1007/s10067-016-3525-1 (PMC5400782; doi:10.1007/s10067-016-3525-1)
Supplement: Supplementary file 1 — (DOCX 19.3 kb) [file 10067_2016_3525_MOESM1_ESM.docx]

**Supplementary Table 1. Population and number of cases of events in first-generation and second-generation in Sweden, 1998-2012**

|  | First-generation | | | | |  | Second-generation | | | | |
| --- | --- | --- | --- | --- | --- | --- | --- | --- | --- | --- | --- |
|  | Population | |  | Gout events | |  | Population | |  | Gout events | |
|  | No. | % |  | No. | % |  | No. | % |  | No. | % |
| Sweden | 5306288 | 82.3 |  | 28900 | 87.7 |  | 5666670 | 82.4 |  | 16287 | 89.4 |
| Denmark | 50363 | 0.8 |  | 227 | 0.7 |  | 80683 | 1.2 |  | 175 | 1.0 |
| Finland | 214001 | 3.3 |  | 1161 | 3.5 |  | 343809 | 5.0 |  | 713 | 3.9 |
| Iceland | 8061 | 0.1 |  | 6 | 0.0 |  | 7663 | 0.1 |  | 1 | 0.0 |
| Norway | 50452 | 0.8 |  | 194 | 0.6 |  | 88551 | 1.3 |  | 232 | 1.3 |
| France | 7063 | 0.1 |  | 16 | 0.0 |  | 7645 | 0.1 |  | 6 | 0.0 |
| Greece | 17127 | 0.3 |  | 44 | 0.1 |  | 20097 | 0.3 |  | 11 | 0.1 |
| Italy | 9481 | 0.1 |  | 52 | 0.2 |  | 12532 | 0.2 |  | 25 | 0.1 |
| Spain | 6939 | 0.1 |  | 17 | 0.1 |  | 8462 | 0.1 |  | 13 | 0.1 |
| Other Southern Europe | 4910 | 0.1 |  | 19 | 0.1 |  | 5841 | 0.1 |  | 5 | 0.0 |
| The Netherland | 7207 | 0.1 |  | 17 | 0.1 |  | 7893 | 0.1 |  | 10 | 0.1 |
| England and Ireland | 20704 | 0.3 |  | 68 | 0.2 |  | 22259 | 0.3 |  | 17 | 0.1 |
| Germany | 40500 | 0.6 |  | 271 | 0.8 |  | 61834 | 0.9 |  | 135 | 0.7 |
| Austria | 6297 | 0.1 |  | 70 | 0.2 |  | 10493 | 0.2 |  | 26 | 0.1 |
| Other Western Europe | 4273 | 0.1 |  | 17 | 0.1 |  | 4856 | 0.1 |  | 4 | 0.0 |
| Bosnia | 37489 | 0.6 |  | 58 | 0.2 |  | 25080 | 0.4 |  | 9 | 0.0 |
| Yugoslavia | 62045 | 1.0 |  | 253 | 0.8 |  | 64930 | 0.9 |  | 70 | 0.4 |
| Croatia | 4729 | 0.1 |  | 24 | 0.1 |  | 2984 | 0.0 |  | 4 | 0.0 |
| Romania | 12172 | 0.2 |  | 46 | 0.1 |  | 6804 | 0.1 |  | 11 | 0.1 |
| Bulgaria | 3995 | 0.1 |  | 7 | 0.0 |  | 2453 | 0.0 |  | 1 | 0.0 |
| Other Eastern Europe | 13479 | 0.2 |  | 10 | 0.0 |  | 3733 | 0.1 |  | 2 | 0.0 |
| Estonia | 9511 | 0.1 |  | 64 | 0.2 |  | 17715 | 0.3 |  | 78 | 0.4 |
| Latvia | 6591 | 0.1 |  | 20 | 0.1 |  | 3765 | 0.1 |  | 16 | 0.1 |
| Poland | 43942 | 0.7 |  | 161 | 0.5 |  | 32128 | 0.5 |  | 55 | 0.3 |
| Other Central Europe | 6306 | 0.1 |  | 45 | 0.1 |  | 8153 | 0.1 |  | 19 | 0.1 |
| Hungary | 13108 | 0.2 |  | 119 | 0.4 |  | 15084 | 0.2 |  | 35 | 0.2 |
| Africa | 64649 | 1.0 |  | 149 | 0.5 |  | 44052 | 0.6 |  | 6 | 0.0 |
| Northern America | 19840 | 0.3 |  | 54 | 0.2 |  | 28290 | 0.4 |  | 65 | 0.4 |
| Chile | 23548 | 0.4 |  | 42 | 0.1 |  | 22078 | 0.3 |  | 8 | 0.0 |
| Southern America | 20661 | 0.3 |  | 37 | 0.1 |  | 14452 | 0.2 |  | 12 | 0.1 |
| Turkey | 36129 | 0.6 |  | 110 | 0.3 |  | 38632 | 0.6 |  | 34 | 0.2 |
| Lebanon | 20431 | 0.3 |  | 46 | 0.1 |  | 19372 | 0.3 |  | 4 | 0.0 |
| Iran | 41758 | 0.6 |  | 118 | 0.4 |  | 30065 | 0.4 |  | 14 | 0.1 |
| Iraq | 72822 | 1.1 |  | 159 | 0.5 |  | 42428 | 0.6 |  | 15 | 0.1 |
| Other Asian countries | 108507 | 1.7 |  | 219 | 0.7 |  | 58134 | 0.8 |  | 38 | 0.2 |
| Russia | 16162 | 0.3 |  | 57 | 0.2 |  | 9776 | 0.1 |  | 22 | 0.1 |
| Others | 57829 | 0.9 |  | 79 | 0.2 |  | 35286 | 0.5 |  | 34 | 0.2 |
| Total | 6449369 | 100.0 |  | 32956 | 100.0 |  | 6874682 | 100.0 |  | 18212 | 100.0 |
