# Supplementary material for: Gout in immigrant groups: a cohort study in Sweden
Source: Clin Rheumatol. 2017 Jan 13;36(5):1091–102. doi: 10.1007/s10067-016-3525-1 (PMC5400782; doi:10.1007/s10067-016-3525-1)
Supplement: Supplementary file 2 — (DOCX 13.8 kb) [file 10067_2016_3525_MOESM2_ESM.docx]

**Supplementary Table 2. Sensitive analysis of risk of gout in the first-generation male immigrants, excluding immigrants who moved to Sweden within 5 years of follow-up***

|  | HR (95% CI) |
| --- | --- |
| Sweden | 1 (ref) |
| **Nordic countries** | **0.87 (0.82-0.93)** |
| Denmark | **0.73 (0.62-0.86)** |
| Finland | 0.94 (0.88-1.02) |
| Norway | **0.71 (0.58-0.86)** |
| **Southern Europe** | **0.71 (0.59-0.85)** |
| France | 0.82 (0.47-1.45) |
| Greece | **0.54 (0.39-0.76)** |
| Italy | 0.89 (0.67-1.19) |
| Spain | 0.59 (0.34-1.02) |
| Other Southern Europe | 0.85 (0.48-1.50) |
| **Western Europe** | **1.13 (1.01-1.26)** |
| The Netherlands | 0.71 (0.41-1.22) |
| UK and Ireland | 1.15 (0.87-1.52) |
| Germany | 1.13 (0.97-1.31) |
| Austria | **1.45 (1.10-1.90)** |
| Other Western Europe | 0.78 (0.42-1.45) |
| **Eastern Europe** | 1.03 (0.92-1.16) |
| Bosnia | 0.82 (0.58-1.16) |
| Yugoslavia | 1.03 (0.90-1.19) |
| Croatia | 1.48 (0.95-2.29) |
| Romania | **1.45 (1.04-2.03)** |
| **Baltic countries** | 0.82 (0.63-1.08) |
| Estonia | 0.79 (0.58-1.07) |
| Latvia | 1.03 (0.55-1.91) |
| **Central Europe** | **1.17 (1.02-1.34)** |
| Poland | **1.28 (1.05-1.55)** |
| Other Central Europe | 1.02 (0.72-1.43) |
| Hungary | 1.11 (0.89-1.38) |
| **Africa** | 1.22 (1.00-1.49) |
| **Northern America** | 0.87 (0.64-1.18) |
| **Latin America** | **0.63 (0.48-0.82)** |
| Chile | **0.51 (0.36-0.74)** |
| South America | 0.83 (0.56-1.23) |
| **Asia** | **1.15 (1.05-1.27)** |
| Turkey | 0.90 (0.72-1.13) |
| Lebanon | 1.04 (0.75-1.44) |
| Iran | 1.20 (0.99-1.46) |
| Iraq | 1.27 (0.97-1.65) |
| Other Asia countries | **1.29 (1.10-1.52)** |
| **Russia** | **1.48 (1.06-2.05)** |

*Adjusted for age, region of residence in Sweden, educational level, marital status, neighborhood deprivation, and comorbidities

HR (95% CI): Hazard ratio with 95% confidence interval
