# Supplementary material for: Gout in immigrant groups: a cohort study in Sweden
Source: Clin Rheumatol. 2017 Jan 13;36(5):1091–102. doi: 10.1007/s10067-016-3525-1 (PMC5400782; doi:10.1007/s10067-016-3525-1)
Supplement: Supplementary file 3 — (DOCX 13.3 kb) [file 10067_2016_3525_MOESM3_ESM.docx]

**Supplementary Table 3. Sensitive analysis of risk of gout in the first-generation female immigrants, excluding immigrants who moved to Sweden within 5 years of follow-up***

|  | HR (95% CI) |
| --- | --- |
| Sweden | 1 (ref) |
| **Nordic countries** | 0.95 (0.87-1.03) |
| Denmark | 0.94 (0.74-1.18) |
| Finland | 0.98 (0.89-1.09) |
| Norway | 0.82 (0.66-1.01) |
| **Southern Europe** | **0.63 (0.41-0.95)** |
| **Western Europe** | 1.09 (0.91-1.30) |
| The Netherlands | 0.87 (0.33-2.32) |
| Germany | 1.02 (0.83-1.27) |
| Austria | **1.71 (1.08-2.71)** |
| **Eastern Europe** | 1.04 (0.83-1.31) |
| Bosnia | 1.29 (0.71-2.34) |
| Yugoslavia | 0.88 (0.66-1.19) |
| Romania | 1.64 (0.91-2.97) |
| **Baltic countries** | 0.87 (0.61-1.25) |
| Estonia | 0.71 (0.47-1.10) |
| **Central Europe** | **1.42 (1.16-1.73)** |
| Poland | 1.26 (0.94-1.67) |
| Other Central Europe | 1.06 (0.60-1.86) |
| Hungary | **1.94 (1.41-2.67)** |
| **Africa** | **2.26 (1.47-3.48)** |
| **Northern America** | **0.37 (0.18-0.77)** |
| **Latin America** | 0.82 (0.52-1.31) |
| Chile | 0.85 (0.48-1.50) |
| **Asia** | 1.14 (0.92-1.40) |
| Turkey | 1.20 (0.84-1.72) |
| Iraq | **1.90 (1.05-3.44)** |
| Other Asia countries | 1.19 (0.85-1.66) |
| **Russia** | 0.74 (0.40-1.38) |

*Adjusted for age, region of residence in Sweden, educational level, marital status, neighborhood deprivation, and comorbidities

HR (95% CI): Hazard ratio with 95% confidence interval
